# Health assessment and the capability approach

**DOI:** 10.1080/11287462.2019.1673028

**Published:** 2019-09-30

**Authors:** Rodrigo López Barreda, Joelle Robertson-Preidler, Paula Bedregal García

**Affiliations:** a Anesthesia Department, Bioethics Center, Faculty of Medicine, Pontificia Universidad Católica de Chile, Santiago, Chile; b Institute of Biomedical Ethics and History of Medicine, Faculty of Medicine, University of Zürich, Zürich, Switzerland; c Public Health Department, Faculty of Medicine, Pontificia Universidad Católica de Chile, Santiago, Chile

**Keywords:** Health evaluation, outcome assessment, process assessment, public policy, ethics

## Abstract

Health has an important role in the achievement of a good quality of life. Many public policies intended to enhance individual and population health. Amartya Sen’s Capability Approach (CA) offers a framework to assess well-being, as well as interventions seeking to increase it. There are, however, important practical challenges that must be faced before applying CA to concrete situations, such as health. One of these challenges is defining whether it is functioning or a capability that is the feature to be assessed. Moreover, some aspects of freedom that are relevant for CA are frequently neglected, such as agency. These aspects must be considered when performing a health assessment using the CA as a framework. A health assessment using the CA as a framework should include indicators based on the achieved dimension (health functioning), resources and conversion factors (health capability), and freedom to achieve (agency).

## Introduction

Health is a central feature for achieving a high quality of life. Health assessment has been a major concern for researchers and scholars, and there is a huge amount of literature on the theoretical and technical aspects of this challenge. Amartya Sen’s Capability Approach (CA) offers a framework to assess human well-being (Anand, Hunter, & Smith, 2005), including health, which has been extensively used in the analysis of public policies and in academic research.

This paper reflects on the assessment of health from a CA perspective, including the distinction between health as a functioning and health as a capability, and the role of agency in this important dimension of human well-being. Firstly, a brief account of the CA and the advantages of using this approach in the context of health are given. Then, the practical challenges of assessing health within the CA approach are discussed and the conceptual shortcomings originated by these proposals. Lastly, the concept of agency is proposed as complementary to capabilities and functionings in order to address some of the difficulties of using the CA in the health context.

### The Capability Approach

The Capability Approach (CA) was developed in 1979 by the Nobel Prize winner Amartya Sen (1979). Since its inception, this approach has received increasing attention by disciplines as varied as economics, political philosophy and political sciences. Sen proposed that human development is not only a matter of achieving and sustaining high rates of economic growth or utilities but also to expand what people are able to do and be – what might be called “real freedoms”. CA considers “a person being able to do certain basic things” (Sen, 1979, p. 218) as the end, and resources and utilities as only means to this end. Furthermore, the CA makes a sharp distinction between achieved functionings, capabilities and resources. Within the CA approach, functionings are the ways of being and doing that a given person has, such as being literate or working (a doing), while capabilities are the bundle of possibilities that a person may transform into an actual functioning (Sen, 1988).

**Figure 1.**
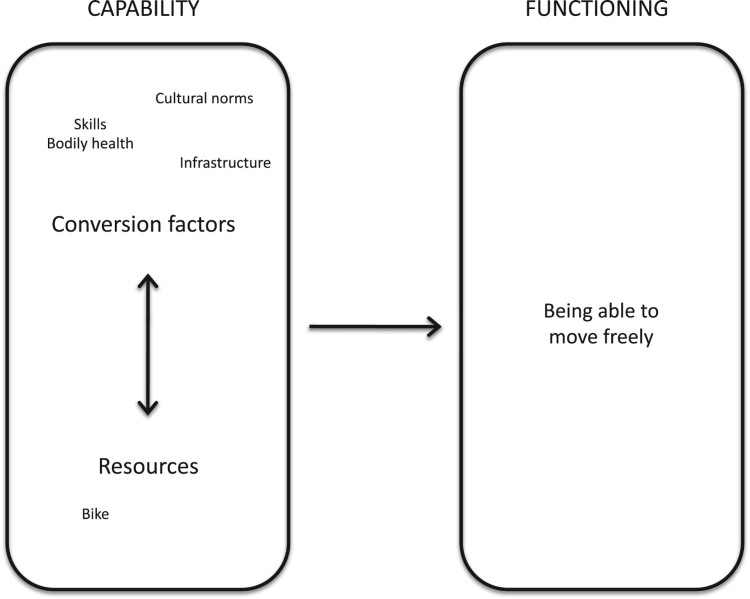
Relationship between capabilities and functionings.

According to this approach, resources and conversion factors can help or impede the ability to transform capabilities into functionings (Robeyns, 2005). For example, the capability to transport oneself to a health care centre can be positively affected by the possession of a bike, a resource. In this example, the ability to ride bikes is a personal conversion factor; the social acceptability of riding the bike is a social conversion factor; finally, the quality of the roads is an environmental conversion factor. Moreover, some functionings could be deemed as personal conversion factors for other capabilities. For example, enjoying good health (a functioning) enhances the capability of mobility, acting as a personal conversion factor. The interaction between conversion factors, resources, and achieved functionings show the complex relationships that underlie capabilities and functionings (Figure 1).

The concepts of functioning, capability, resources and conversion factors have been analysed theoretically, but they have also been used in practice to develop measurement tools aiming at assessing individual and collective well-being. Many frameworks take into account resources and material goods in the assessment of people’s living conditions, some approaches reaching the point of almost equalizing the Gross Domestic Product (GDP) with the population’s level of well-being. Other frameworks consider human basic needs, basic liberties and/or primary goods as proxies to assess welfare, but the specificity of the CA is that it is the only approach that takes personal variability into account as it acknowledges that individuals may differ in their ability to transform resources into actual well-being. It could be said that while other theories highlight the importance of opportunities, the CA is concerned with genuine opportunities, as under some circumstances, an option is only theoretically attainable for a given individual (Wolff & de-Shalit, 2007). One of the most famous practical applications of the CA is the Human Development Index (HDI) developed by the UNDP.

## Health in CA

The CA is a framework that delves into the interactions between resources, capabilities and functionings, acknowledging that individuals with different preferences, needs and conversion factors may require personalized support and tailored resource use to achieve valuable goals (Robeyns, 2005). It is said that the CA “is deeply concerned with the effects of … unequal opportunity, and concentrates on personal capacities and interpersonal ties … ” (Latham, 2012, p. 127). This particular sensitivity to personal variability makes this approach a very compelling framework to apply in the case of health, as people with particular conditions may achieve the same health functioning as others, but demand a more intensive use of health services. For example, a healthy child requires regular check-ups, some prevention interventions (such as vaccines) and eventual health care interventions to stay healthy, but a child suffering from some metabolic diseases may enjoy a similar quality of life, but at the expense of significant healthcare resources. Hence, the CA seems to be a reasonable framework to address health issues.

There are some theoretical issues still to be defined in CA when assessing health, such as the fact that health has an objective (observable) dimension, but also a subjective (perceivable) dimension. As Sen points out (Sen, 2002), the observable dimension is almost always taken into account when assessing health, but the self-perceived character of health should not be neglected. In any case, health is generally deemed a fundamental feature for human well-being, and is often considered one of the basic capabilities, a category reserved for “abilities to satisfy certain elementary and crucially important functionings up to certain level” (Sen, 1992, p. 45).

There are also important practical issues to be resolved when assessing capabilities and functionings (Robeyns, 2005). This is also the case for health, which can be regarded as a capability (the effective opportunity of achieving a given level of health) or as a functioning (the achieved level of health). There is still no consensus among the CA scholars whether the assessment has to be performed on the capability dimension or the functioning dimension. Moreover, the way to operationalize both, health functioning and health capability, has not been defined so far. This is why it has been claimed that “one problem with the capability approach is that of identifying suitable empirical measures which can be used in its support with the result that its relevance has been questioned” (Anand, Hunter, et al., 2005, p. 13)

### Health as a functioning

When assessing health, analysing concrete functioning instead of the corresponding capability has many advantages. Firstly, functionings are easier to appraise because they are achieved features with actual characteristics, and not potential conditions. Nutritional state, for example, can be roughly measured using cheap and available techniques (anthropometry) or simple laboratory tests; on the other hand, the capability of being well nourished requires an estimation of the availability of and access to food, which in turn are related to economic, social and cultural characteristics (such as time to eat and feeding priorities). Secondly, it has been argued that people tend to have the same preferences regarding the most basic personal needs, such as health. If all people have the same priorities regarding these needs and the same effective opportunities to meet them, the distribution of achievements will be similar, and it would follow that assessing real conditions (functionings) will be a sensible and feasible task. Lastly, under the assumption that health is a necessary condition for flourishing and lack of health is deemed a corrosive disadvantage (Wolff & de-Shalit, 2007), the functioning of being healthy should be considered a desirable outcome.

In the CA literature much research analyses health as a functioning. Several proxies for health have been proposed in terms of the absolute absence of health, such as mortality rate, infant mortality rate and under-five mortality rate (Hausman, Asada, & Hedemann, 2002; McGuire, 2010). Other constructs like morbidity indexes, disability-free life expectancy and health expectancy are focused on the relative absence of health (Hausman et al., 2002). There are also indicators based on the impact of health on quality of life, e.g. quality-adjusted life years or QALY. Finally, studies in the CA literature have treated health as functioning in order to describe its effect on subjective well-being (Anand, Santos, & Smith, 2007).

Assessing the functioning has the advantage of accuracy and comparability, and also the fact that it allows for the appraisal of interactions between health and other capabilities, but the disadvantage of overlooking personal variability and personal preferences. Moreover, the myriad existing indicators to measure and assess health impose the challenge of selecting and weighting the relevant indices to obtain a comprehensive evaluation of people’s health. Without such a task, these indicators may lead to incomplete results or be difficult to interpret.

### Health as a capability

A frequently used argument for focusing on capabilities instead of functionings is that functionings only take outcomes into account, which can neglect the opportunities that people have to achieve these assumed goals (Ruger, 2010). How should the “genuine opportunity” of achieving good health be appraised?

Health status depends on personal factors, such as individual predisposition and lifestyle, health care, and the social determinants of health, or “the conditions in which people live their daily lives and the structural influence on these conditions that ultimately reflect the distribution of power and resources” (World Health Organization, 2012, p. 9). Among the most commonly cited social determinants of health are structural determinants, such as political institutions, and intermediate determinants, such as income, educational level, housing, neighbourhood, and working conditions (World Health Organization, 2012). These determinants have been organized in many different models, aiming at identifying links between social determinants and health inequalities (Borrel, Malmusi, & Muntaner, 2017). These models range from linear in shape (Mosley & Chen, 1984) to more organic and complex in structure (House & Williams, 2003; Kim & Saada, 2013), including those taking into account geographical and temporal variables (Marmot, 2010). The similarities between the concepts of the social determinants of health and the aforementioned social and environmental conversion factors lead to the idea that through the analysis of the social determinants of health is possible to assess health as a capability (Hall, Taylor, & Barnes, 2013).

Assessing health based exclusively on its determinants may yield complex conclusions. Paradigm for the analysis may vary in different scenarios and the data and conclusions from one study are not necessarily applicable to other contexts. For example, female illiteracy is associated to the infant mortality rate in low-income countries, but this association does not occur in high-income countries (Schell, Reilly, Rosling, Peterson, & Ekström, 2007). Moreover, the social determinants of health literature focus on health inequalities among social groups and not necessarily in how social categories affect health at an individual level. It has been claimed “the causal mechanisms linking social rank to poor health are not always well specified” (Hall et al., 2013, p. S178). This is a very relevant issue for CA literature, especially when normative claims are based on empirical data. Hence “without a solid understanding of causality, we will not be able to say with confidence what causes capabilities to go up or down, flourish or fail” (Venkatapuram, 2011, p. 238). Furthermore, the meaning of health capability itself (and its connection to social determinants of health) has not properly been defined so far, making the normative analysis even more problematic (Mitchell, Roberts, Barton, & Coast, 2017).

### Assessing health using the CA framework: a missing dimension

As was commented beforehand, a major challenge of applying the CA approach to specific situations, including health, is that there is no consensus about whether the functioning or the capability should be examined (Robeyns, 2005). Making this definition is not an easy task since both approaches have shortcomings and advantages. It has been claimed that “in its present state the capabilities approach does not have the tools to make it easily operational” (Venkatapuram, 2011, p. 238). In fact, both health functioning and health capability are important components in the assessment of health in a comprehensive way, and no single indicator can provide a full picture of health (Powers & Faden, 2006).

There is, however, an often-missing component of CA, which is an explicit inspection of people’s abilities and attitudes towards transforming their capabilities into functionings.

### Health agency

Let us consider that periodic check-ups for infants reduce child mortality, and a public policy that increases the availability of heath care practitioners to provide this service is implemented. It could be said that the capability dimension was increased by this public policy, but if there are cultural barriers and parents do not attend health care facilities, the functioning dimension would remain unchanged. Making regular infant check-ups compulsory would most likely increase health functioning assessed in terms of child mortality, but would limit families’ freedom and their capacity to make decisions regarding health care.

There is another feature of the CA that must be highlighted, and which is frequently neglected in empirical assessments. This approach focuses on people’s capabilities to function in order to increase their opportunities to “live the kind of life that they have a reason to value” (Robeyns, 2005, p. 94). The fact that people should value their own achievements expands the scope of well-being beyond functionings, resources and opportunities, encompassing people’s attitudes and ability to pursue and achieve goals (Sen, 1999). People, then, should not only enjoy valuable features, such as good health but also value the goal and be involved in the process of bringing about this goal. Agency understood as “the ability to act on behalf of what you value and have a reason to value” (Ibrahim & Alkire, 2007, p. 383) is a concept which may be used to address this dimension of well-being. Someone with this ability – to act on behalf of what he/she values – would be an agent. Hence, CA acknowledges that inputs and outcomes cannot be the sole dimension of well-being, but the process that brings about achievement must be analysed as well. Ruger echoes this issue when she proposes an approach to health capability which includes health agency, i.e. “individuals’ ability to achieve health goals they value and act as agents of their own health” (Ruger, 2010, p. 42). Freedom to achieve is at the core of CA and any operationalization of health should consider it as a relevant feature. Assessing only objective outcomes and designing interventions merely based on functionings may incur the risk of paternalism, as these outcomes can be brought about by means of indoctrination, nagging and even coercion (Ruger, 2010).

The link between agency, capabilities and functionings is an issue of forgoing discussion. For some authors, “resources and agency together constitute what Sen refers to as capabilities” (Kabeer, 1999, p. 438), making agency a component of capabilities; others have identified agency with two capabilities from the well-known Martha Nussbaum’s list, namely practical reason and affiliation (Nussbaum, 2000). The complexity of this issue lies in the different nature of these concepts: capability and functioning refer to particular ways of being and doing, either as achieved characteristics (functioning) or as a potential feature (capability); agency, on the other hand, is a specific mode of acting which presupposes some degrees of valuing and reasoning. For instance, being literate is a way of being, hence a functioning; if a person values this skill and employs it for the sake of something she values, reading would be an *agentive* performed action.

Since agency is a way of doing, it is indeed functioning, and the freedom to deploy this specific way of acting would be the capability of agency. Then, is agency a component of capabilities (Kabeer, 1999) or a functioning in itself (Nussbaum, 2000)? There is no contradiction between these accounts, since “some ends are simultaneously also means to other ends (e.g. the capability of being in good health is an end in itself, but also a means to the capability to work)” (Robeyns, 2005, p. 95). In other words, capabilities often require another functioning to exist. For example, the functioning of good health enhances the capability of mobility, as was commented beforehand.

Agency is a relevant element for assessing health using CA as a framework, and can feasibly be measured using Self-Determination Theory (SDT) (Deci, 1971), as was proposed by Alkire (2005). This theory allows for the assessment of agency using validated empirical surveys across different dimensions, such as education, healthcare, work and housing and different cultural contexts. Some surveys have already followed this suggestion and used agency to study capabilities and functionings (International Food Policy Research Institute, 2012; Pallai & Alkire, 2007). Agency is then a measurable concept. Since it informs the ability to act according to personal values, it accounts for the missing dimension when assessing health within CA.^1^ Moreover, by means of agency, it is possible to integrate some accounts of the subjective dimension of health, to which Sen referred when making the distinction between objective and subjective dimensions of health (Sen, 2002).

This account of agency has been introduced in biomedical ethics (López, Trachsel, & Biller-Andorno, 2016) and it has consequences at collective and individual levels. Public health policies should consider agency not only during the assessment stage but also during design and implementation. This is a reasonable alternative, which might decrease the risk of developing well-intended, but paternalistic policies, in which patients are “passive recipients of interventions designed to meet this goal” (Levine, 2013, p. 55). It has to be admitted that people’s agency may vary according to their personal circumstances. Public policies should enhance people’s agency as much as possible, but there are cases when individuals cannot exercise an adequate level of agency. Then, it would be possible to implement more directive policies, aiming at the protection of vulnerable people. At an individual level, this task would foster the so-called patient-centred care (Levine, 2013) and the support for self-management (Entwistle, Cribb, & Owens, 2018). At a collective level, it should be considered that creating public policies using the CA as a framework “emphasizes deliberatively derived public policy for human flourishing and reasoned consensus to evaluate arrangements for improving human functioning” (Ruger, 2006, p. 157). A reasonable requisite to fulfil agency demands at the collective level is a popular deliberation process which includes all the stakeholders.

## Conclusion

This account has some implications regarding assessment and interventions in health using the CA as a framework. There are sound arguments to assess health as a functioning and very valid arguments to evaluate health as functioning. Nevertheless, any attempt to assess health based on only one of these dimensions may provide a partial account. Hence, a health assessment from the CA perspective should include indicators based on the achieved dimension (health functioning), and resources and conversion factors (health capability). However, as freedom to achieve is an important constituent of CA, including attitudes, aims, values and involvement, and any attempt to assess health using CA as a framework should take into account some measurement of this factor (agency).

An appropriate health indicator reflecting the population’s achieved health status could be under-5 mortality since it is mostly related to living conditions (McGuire, 2010). Examining the social determinants of health, such as education, housing, employment and/or economic inequalities, may help to provide a better picture of resources, social and environmental conversion factors. Even though it may be claimed that agency is difficult to assess, some empirical tools derived from Self-Determination Theory have been identified and successfully used to examine agency in real scenarios, making this challenge a feasible task.

## Note

1 More comprehensive interpretations of the social determinants of a health framework, such as Nancy Krieger’s work (32), acknowledge that social factors affect people’s ability to exert control over their lives; then, the social determinants of health are more than mere resources and opportunities. Under this interpretation, agency would not be a necessary construct as it would be shaped by the aforementioned social factors. There is an important distinction, however, that must be highlighted: while the social determinants of health examine social factors from a group perspective, the agency concept focuses on individuals’ internal ability to exert control over their lives; agency may be affected by external factors, but it gives room to interindividual variability across social groups.

## References

[CIT0001] Alkire S. (2005). Subjective quantitative studies of human agency. *Social Indicators Research*, 74, 217–260. doi: 10.1007/s11205-005-6525-0

[CIT0002] Anand S., Hunter G., & Smith R. (2005). Capabilities and well-being: Evidence based on the Sen–Nussbaum approach to welfare. *Social Indicators Research*, 74, 9–55. doi: 10.1007/s11205-005-6518-z

[CIT0003] Anand S., Santos C., & Smith R. (2007).*The measurement of capabilities* (Open Discussion Papers in Economics, No 67). London: The Open University.

[CIT0004] Borrel C., Malmusi D., & Muntaner C. (2017). Introduction to the “evaluating the impact of structural policies on health inequalities and their social determinants and fostering change” (SOPHIE) project. *International Journal of Health Services*, 47, 10–17. doi: 10.1177/0020731416681891 27956577

[CIT0005] Deci E. L. (1971). Effects of externally mediated rewards on intrinsic motivation. *Journal of Personality and Social Psychology*, 18, 105–115. doi: 10.1037/h0030644

[CIT0006] Entwistle V. A., Cribb A., & Owens J. (2018). Why health and social care support for people with long-term conditions should be oriented towards enabling them to live well. *Health Care Analysis*, 26, 48–65. doi: 10.1007/s10728-016-0335-1 27896539 PMC5816130

[CIT0007] Hall P. A., Taylor R. C. R., & Barnes L. (2013). A capabilities approach to population health and public policy-making. *Revue D’Epidemiologie et de Sante Publique*, 61(S3), S177–S183. doi: 10.1016/j.respe.2013.05.016 23835148

[CIT0008] Hausman D. M., Asada Y., & Hedemann T. (2002). Health inequalities and why they matter. *Health Care Analysis*, 10, 177–191. doi: 10.1023/A:1016502119050 12216744

[CIT0009] House J. S., & Williams D. R. (2003). Understanding and reducing socioeconomic and racial/ethnic disparities in health. In Hofrichter R. (Ed.), *Health and social justice: Politics, ideology, and inequity in the distribution of disease* (pp. 89–131). Hoboken: Jossey-Bass.

[CIT0010] Ibrahim S., & Alkire S. (2007). Agency and empowerment: A proposal for internationally comparable indicators. *Oxford Development Studies*, 35, 379–403. doi: 10.1080/13600810701701897

[CIT0011] International Food Policy Research Institute (2012). *Women’s empowerment in agricultural index*. Washington: The U.S. Government’s Global Hunger & Food Security Initiative.

[CIT0012] Kabeer N. (1999). Resources, agency, achievements: Reflections on the measurement of women's empowerment. *Development and Change*, 30, 435–464. doi: 10.1111/1467-7660.00125

[CIT0013] Kim D., & Saada A. (2013). The social determinants of infant mortality and birth outcomes in western developed nations: A cross-country systematic review. *International Journal of Environmental Research and Public Health*, 10, 2296–2335. doi: 10.3390/ijerph10062296 23739649 PMC3717738

[CIT0014] Krieger N. (2014). Discrimination and health inequities. *International Journal of Health Services*, 44, 643–710. doi: 10.2190/HS.44.4.b 25626224

[CIT0015] Latham S. R. (2012). Justice of and within health care finance. In Rhodes R., Battin M. P., & Silvers A. (Eds.), *Medicine and social justice; essays on the distribution of health care* (2nd ed.) (pp. 121–130). New York, NY: Oxford University Press.

[CIT0016] Levine R. L. (2013). Disabling the patient by incorporating capabilities approach into person-centred care. *The American Journal of Bioethics*, 13, 55–56. doi: 10.1080/15265161.2013.802070 23862606

[CIT0017] López R., Trachsel M., & Biller-Andorno N. (2016). Towards a broader understanding of agency in biomedical ethics. *Medicine, Health Care and Philosophy*, 19, 475–483. doi: 10.1007/s11019-016-9706-5 27142686

[CIT0018] Marmot M. (2010). *Strategic review of health inequalities in England post-2010: Fair society, healthy lives – the Marmot review. Executive summary*. London: The Marmot Review.

[CIT0019] McGuire J. W. (2010). *Wealth, health, and democracy in East Asia and Latin America*. New York, NY: Cambridge University Press.

[CIT0020] Mitchell P. M., Roberts T. E., Barton P. M., & Coast J. (2017). Applications of the capability approach in the health field: A literature review. *Social Indicators Research*, 133, 345–371. doi: 10.1007/s11205-016-1356-8 28769147 PMC5511308

[CIT0021] Mosley W. H., & Chen L. C. (1984). An analytical framework for the study of child survival in developing countries. *Population and Development Review*, 10, 25–45. doi: 10.2307/2807954 PMC2572391 12756980

[CIT0022] Nussbaum N. (2000). *Women and human development*. New York, NY: Oxford University Press.

[CIT0023] Pallai V., & Alkire S. *Measuring individual agency or empowerment: A study in Kerala*. Kerala, India: Centre for Development Studies Thiruvananthapuram; 2007.

[CIT0024] Powers M., & Faden R. (2006). *Social justice*. New York, NY: Oxford University Press.

[CIT0025] Robeyns I. (2005). The capability approach: A theoretical survey. *Journal of Human Development*, 6, 93–117. doi: 10.1080/146498805200034266

[CIT0026] Ruger J. P. (2006). Health, capability, and justice: Towards a new paradigm of health ethics. *Policy and Law. Cornell Journal of Law Public and Policy*, 15, 101–187. 17136814

[CIT0027] Ruger J. P. (2010). Health capability: Conceptualization and operationalization. *American Journal of Public Health*, 100, 41–49. doi: 10.2105/AJPH.2008.143651 19965570 PMC2791246

[CIT0028] Schell C. O., Reilly M., Rosling H., Peterson S., & Ekström A. M. (2007). Socioeconomic determinants of infant mortality: A worldwide study of 152 low-, middle-, and high-income countries. *Scandinavian Journal of Public Health*, 35, 288–297. doi: 10.1080/14034940600979171 17530551

[CIT0029] Sen A. (1979). *Equality of what? The Tanner lecture on human values*.

[CIT0030] Sen A. (1988). Freedom of choice. *European Economic Review*, 32, 269–294. doi: 10.1016/0014-2921(88)90173-0

[CIT0031] Sen A. (1992). *Inequality re-examined*. Cambridge: Harvard University Press.

[CIT0032] Sen A. (1999). *Development as freedom*. New York, NY: Oxford University Press.

[CIT0033] Sen A. (2002). Health: Perception versus observation. *British Medical Journal*, 324, 860–861. doi: 10.1136/bmj.324.7342.860 11950717 PMC1122815

[CIT0034] Venkatapuram S. (2011). *Health justice*. Cambridge: Polity Press.

[CIT0035] Wolff J., & de-Shalit A. (2007). *Disadvantage*. New York, NY: Oxford University Press.

[CIT0036] World Health Organization (2012). *Addressing the social determinants of health: The urban dimension and the role of local government*. Denmark: WHO – Regional Office for Europe.

